# Animal Abuse Investigations: Challenges and Recommendations to Improve Animal and Human Welfare

**DOI:** 10.3390/ani14243602

**Published:** 2024-12-13

**Authors:** Rebecca Niemiec, Lori R. Kogan

**Affiliations:** 1 CSU Animal-Human Policy Center, Colorado State University, Fort Collins, CO 80523, USA; rebecca.niemiec@colostate.edu; 2 Clinical Sciences Department, Colorado State University, Fort Collins, CO 80523, USA

**Keywords:** animal abuse, animal neglect, law enforcement, trauma, animal protection, animal law

## Abstract

Animal cruelty is widespread, yet it is often overlooked by professionals as a form of trauma and not perceived as a serious crime. This study explores current challenges to the effective management of animal abuse and neglect cases in the state of Colorado, USA, to better understand systemic barriers and their impact on those involved, and offers concrete, practical suggestions for improvement. Interviews with 24 law enforcement and Bureau of Animal Protection (BAP) agents/staff were conducted, and an integrated thematic analysis method was utilized to inductively analyze participants’ responses to interview questions. The most common barriers to addressing animal cruelty cases identified by law enforcement/BAP personnel included (1) a lack of places to take removed animals and resources to care for them; (2) challenges working with the district attorney’s office; (3) a general lack of knowledge and training on how to address cases and the documentation required; and (4) insufficient enforcement personnel to conduct investigations and conduct required follow-up. Suggested changes include expansion of temporary housing options, additional training, expert investigators and social workers/mental health professionals, and possible amendments to state statutes. In addition, the use of a trauma-informed model is recommended to best meet the psychological needs of all those involved in animal welfare work.

## Simple Summary

Many people do not realize how common animal cruelty is. Even professionals often do not see it as a serious crime or understand how traumatic it can be. This study looked at problems with handling animal abuse and neglect cases in the state of Colorado, USA, to determine challenges and possible solutions. The researchers interviewed 24 law enforcement and animal protection workers. Those interviewed shed light on four main problems they face when dealing with animal cruelty: not enough places to keep rescued animals or resources to care for them; difficulties working with district attorneys on these cases; not enough training on how to handle abuse cases; and not enough people to investigate cases and conduct follow-up. Suggested solutions include creating more temporary homes for rescued animals, providing better training for workers, hiring special investigators and mental health experts, and changing state laws. Recommendations also include using methods that consider the impact of trauma of those involved in animal welfare work.

## 1. Introduction

Animal cruelty has received growing research attention over the past few decades, in part due to the associations found with interpersonal violence [1,2]. This interest led, among other things, to the Federal Bureau of Investigation (FBI) adding animal cruelty offenses to its collection of incident-based crime statistics in the Uniform Crime Reporting (UCR) Program’s National Incident-Based Reporting System (NIBRS) in 2014. This inclusion, after years of effort, offers new opportunities to better understand animal cruelty and neglect crimes and address gaps in the research [3].

Far from unusual, it appears that animal cruelty is widespread [4]; one recent study found that over 50% of US college students have intentionally harmed or killed at least one animal [5]. Additionally, NIBRS data from 2018 suggest there were 4.43 animal cruelty incidents nationally per 100,000 people, compared to 106.68 for assault and 65.77 for robbery [6]. Yet animal cruelty is often overlooked by professionals as a form of trauma [7] and not perceived as a serious crime [8,9].

Crimes require legislation, and animal welfare and cruelty legislation is the primary way in which to promote animal welfare and deal with acts of cruelty [10,11]. Every state in the United States has laws around animal cruelty, but these laws can vary widely, including in their definition of cruelty, enforcement, and penalties. To help with enforcement and prosecution efforts, training materials on animal cruelty have been made and disseminated to law enforcement and prosecuting attorneys by several organizations including the National District Attorneys Association, the Association of Prosecuting Attorneys, the International Association of Chiefs of Police, the National Sheriff’s Association, and the U.S. Department of Justice’s Community Oriented Policing Services office [12].

Despite these educational efforts, however, prior research has suggested that many law enforcement agencies and agents feel that animal abuse is not within their sphere of responsibility or that these cases are a low priority [13]. In addition, many officers who investigate animal-related complaints feel undervalued within the law enforcement hierarchy [14]. However, dealing with animal abuse and neglect cases not only involves risk of physical harm, but emotional distress and role strain as officers often must act as both law agents and mental health professionals [15,16,17].

Compassion fatigue, documented in numerous helping professions, is a common factor for law enforcement agents [18,19,20]. These professionals report frequent exposure to traumatic experiences and burdens related to the ‘cost of caring’, both of which negatively impact mental health [18,21,22,23]. The cost of caring extends to animals and many animal welfare workers struggle with repeated exposure to animal suffering [15,17,20,24]. Despite this, only recently has research begun to explore the prevalence of compassion fatigue among professionals involved with animal welfare [25,26,27,28,29].

Yet, viewed through the One Welfare concept, the well-being of both animals at risk for abuse and those working within the animal welfare sector are deserving of attention. The One Welfare concept emphasizes the interconnection between animal and human well-being and recognizes the impact that witnessing animal abuse and neglect can have on animal welfare workers [20,30]. It is therefore important to identify challenges to adequately address animal abuse and neglect cases while also recognizing the psychological impact on those dealing with these cases. To this end, this study was designed to assess the challenges in effectively addressing cruelty and neglect cases as well as the emotional toll associated with working in this field. Because Colorado has comprehensive state resources to enforce animal cruelty laws and is ranked among the top five states in the US by the Animal Legal Defense Fund’s Annual Animal Protection Laws Rankings Report in terms of animal protection laws [8], it was chosen as an ideal case study. Even in states with relatively robust animal protection laws and systems, there is room for improvement. Therefore, our study explores current challenges to the effective management of animal abuse and neglect cases in Colorado to better understand systemic barriers and their impact on those involved and offers concrete, practical suggestions for improvement.

This case study is part of a larger project assessing the views of law enforcement and Bureau of Animal Protection (BAP) agents/staff. In Colorado, there are a variety of resources that exist to help enforce the state’s cruelty statute (for more details about Colorado’s civil and criminal laws addressing animal cruelty and neglect, see Supplementary S1). The Bureau of Animal Protection within the Department of Agriculture is a program dedicated to assisting law enforcement and applying civil remedies to enforce the provisions of the state’s Animal Protection Act. The BAP program has a manager, veterinarian, and multiple investigators who can assist law enforcement with investigations and animal removal and enforce civil remedies focused on animal mistreatment. The BAP also oversees and trains approximately 100 commissioned volunteer animal protection agents from municipalities, law enforcement, and non-profits. These agents can conduct investigations related to animal mistreatment and issue summons and complaints, typically in partnership with local law enforcement. The Colorado Humane Society (CHS), a program of the Dumb Friends League, is one non-profit organization that employs several BAP agents who assist law enforcement throughout the state on companion animal investigations and removal (including equines). While the number of full-time staff at the BAP program employed by the Colorado Department of Agriculture has been expanding since 2022, the number of non-profit, law enforcement, or municipal BAP animal protection agents has fluctuated due to organizational changes. These organizational changes, and the need to inform strategic development of programs to help inform animal cruelty/neglect cases, were the impetus for this study.

The BAP program is what makes Colorado’s approach to enforcing animal cruelty laws one of the more robust, as it provides state-wide resources to help with enforcement of cruelty laws. While Colorado is the only state employing numerous animal cruelty-focused staff within the state’s Department of Agriculture, several other states have developed programs for ensuring there are experts available to assist with animal cruelty cases, typically via partnerships with non-profit organizations. The state of Oregon, for example, allocates funds from the state budget to maintain the Animal Cruelty Deputy District Attorney (AC-DDA) role, which is a special prosecutor dedicated to pursuing animal-related cases. The non-profit Oregon Humane Society employs Humane Special Agents, which are highly trained, sworn police officers with authority to investigate allegations of animal cruelty crimes within the State of Oregon (https://www.oregonhumane.org/report-cruelty/ accessed on 15 November 2025). The program also has a social worker [31] and forensic veterinarian and animal crime forensics center [32] dedicated to these types of cases. In California, non-profit spcaLA Humane Officers may exercise the powers of a peace officer anywhere in the state while investigating animal cruelty. Similarly to Colorado’s BAP agents, these Humane Officers are appointed by the state and undergo substantial training in order to investigate cruelty cases and prosecute individuals or organizations who neglect or abuse animals anywhere within the state of California [33].

In this study, we report results pertaining to barriers and resource needs perceived by law enforcement officers and Bureau of Animal Protection (BAP) agents/staff to being able to investigate and effectively address cases of animal cruelty and neglect. In addition, we highlight sentiments reflecting personal distress in the form of helplessness, frustration and moral injury to better understand how the challenges related to cases of animal cruelty and neglect affect the mental health and well-being of these professionals.

## 2. Materials and Methods

We conducted 24 semi-structured interviews with law enforcement and Bureau of Animal Protection (BAP) agents/staff (see Supplementary S2 for interview questions). Following the principals for qualitative interviews outlined by McGrath et al., [34] the interview guide was created to be flexible, allowing for appropriate follow-up questions or clarifications. Interview questions were created and then piloted to ensure understanding and inclusiveness of all key elements. Care was taken during the interviews to ensure that the interviewers did not influence or bias participants’ responses. The interviews occurred between July and August 2024: eighteen with leadership in a law enforcement agency, including sheriffs, undersheriffs, sergeants, lieutenants, commanders, and deputies; one interview with a deputy who worked animal cases for their jurisdiction; three interviews with BAP staff; and two interviews with Colorado Humane Society staff (who were non-profit BAP agents who assisted law enforcement with companion animal and equine investigations). All interviews took place over Zoom except two that took place in-person at a location selected by the interviewee and lasted between 30 and 75 min.

Law enforcement/BAP interviewees, geographically representative of the whole state of Colorado, were recruited via direct email through personal contacts of a BAP agent who assisted with the study and one of the authors (BN) through their experience working with BAP and law enforcement. We recruited all three Colorado Department of Agriculture Bureau of Animal Protection staff members who conduct civil investigations and assist with criminal investigations throughout the state as well as all three Colorado Humane Society Bureau of Animal Protection agents who assist law enforcement in 44 different jurisdictions throughout the state. We also recruited law enforcement officers from 44 law enforcement agencies who have a Memorandum of Understanding to work with the Colorado Humane Society Bureau of Animal Protection agents on animal cruelty/neglect cases, as well as 1 additional agency that has partnered with the Colorado Department of Agriculture Bureau of Animal Protection on cases. The law enforcement jurisdictions contacted were primarily in rural counties spanning all geographic areas of the state. We contacted the sheriff and undersheriff of each jurisdiction; agencies then determined who would be present at the interview.

### Analyses

We used an integrated thematic analysis method [35] to inductively analyze participants’ responses to the interview questions. Thematic analysis requires that researchers approach data without predetermined themes, instead through the course of analysis, to determine themes based on participants’ stated experience with a specific phenomenon [36]. All interviews were transcribed using NVivo (version 14) transcription software and qualitative thematic analysis was conducted on all transcriptions. Each interview question response was coded into inductive themes using NVivo software. After all interviews were coded, themes were examined in NVivo and similar sub-themes were combined into larger themes. To enhance trustworthiness, the research team maintained an audit trail, tracking the processes throughout the data analysis. Analytic memos reflected decisions surrounding code names, emerging themes, and saturation in the data [37]. The team noted when new data no longer elicited novel codes (code saturation) or elaborated on meanings for the codes (meaning saturation) [37]. Additionally, the audit trail helped structure team discussions about patterns in the data and nuanced meanings within and between questions. To protect the identity of study participants, we kept their anonymity but included their position (i.e., sheriff, undersheriff, sergeant, lieutenant, commander, deputy) and their participant code.

## 3. Results

A total of 24 semi-structured interviews were conducted. These included five interviews with investigators from BAP, six interviews with law enforcement officers, three interviews with law enforcement officers in a leadership role, nine interviews with law enforcement sheriffs, and one interview with a law enforcement undersheriff (Table 1). Analysis of the interviews resulted in three major themes: barriers, needed additional resources, and the emotional toll on participants.

### 3.1. Barriers to Addressing Animal Cruelty and Neglect Cases

The most common barriers to addressing animal cruelty cases identified by law enforcement/BAP included (1) a lack of places to take removed animals and resources to care for them; (2) challenges working with the district attorney’s office; (3) a general lack of knowledge and training on how to address cases and the documentation required; and (4) insufficient enforcement personnel to conduct investigations and conduct required follow-up. We summarize each of these below with illustrative quotes.

#### 3.1.1. Lack of Places to Take Removed Animals and Resources to Care for Them

One of the most common challenges discussed in law enforcement interviews was a lack of places to take removed animals and resources to care for them. This challenge was especially prevalent for livestock, but for some agencies, it also included companion animals. Interviewees reported struggling to find care for livestock in critical condition and being forced to euthanize or leave birds for a lack of safe places to take them. In some instances, law enforcement agents reported housing animals in their own home and paying for the costs of care out of their personal money due to lack of resources.

*“[For] a large animal [we] will try to figure something out to get them out. But then we’re kind of left with the idea of where do we put these animals ? We’re not set up in fairgrounds. We’re not set up in any place to where we can physically bring large animals… But the seizure of the animal is likely not going to happen because we don’t have the resources to place it.”*

[Commander B]

*If they’re being removed from a property, there’s really not a lot of good options for like emergent medical care for livestock. Very often they are seen as a commodity. And so, you know, it’s kind of a cost benefit thing. And so often times we’ll send them to the sale barn […] and their fate is unknown at that point.*

[Investigator—BAP staff/agent, F, #7]

*“We found a situation where animals are experiencing pain and suffering because of the way they’ve been housed or their lack of food or water or whatever. […] Where do we take them ? I have been in multiple situations where I’ve been on properties, I’ve deemed the situations to be inhumane and animals have to be removed and I have to leave the birds there because we have nowhere to take the birds. And that’s happened. I can think right off the top of my head three times that I have left birds because I can’t take them and every other animal has been removed from the property.”*

[Investigator—BAP staff/agent, F, #7]

*“I feel like having lack of housing, lack of resources, is really what we’re running into with all of our cases and trying to find suitable holding facilities or shelters or sanctuaries or something like that. And now more than ever […], we’re having to depopulate chickens because of HPAI. And we can’t find any suitable places for any of these birds we’re moving. We would like to not take them to sale barns or take them to places where they don’t really have a fighting chance, I guess.”*

[Investigator—BAP staff/agent, F, #8]

*“We have to depend on the city’s animal shelter. And I may or may not get those dogs in there. It just depends on which way the wind is blowing that day, because our office does not have a facility to take animals to […]”*

[Law enforcement officer, M, #11]

*“They don’t have any shelters in rural Colorado. And if they do, they’re so small and they’re already overran. You know, they already they’re full. So they don’t have any place to take these animals.”*

[Investigator—BAP staff/agent, F, #1]

*“I think one of our big issues in our area is animal impounds for the small animals. We just we don’t have it here.”*

[Law enforcement officer, M, #11]

*The scramble of where do we place these animals is one of the more difficult things that we face.”*

[Law enforcement officer/leadership, M/M, #21]

#### 3.1.2. Challenges with District Attorneys

Interviewees commonly mentioned challenges working with district attorneys on cases; specifically, interviewees mentioned a lack of communication between the DA’s office and law enforcement and DAs dropping charges or not addressing cases correctly because of a perceived lack of understanding of these types of cases.

*“But it was a distressing case to know that those animals were returned to that owner because of a weak case built in courts and points where it fell apart on ownership establishment. And I think there were some other things that just didn’t fly. I think the DA didn’t have a full understanding of what the medical concerns were and maybe wasn’t experienced in building a cruelty case. And I don’t think the resources were there for the D.A. to feel that maybe they had the support they needed. And, you know, the case fell apart, I think, at multiple points.”*

[Investigator—BAP staff/agent, F, #4]

*“We can put together the most amazing case and send it to our sheriff’s office. But then there seems to be like a… lack of communication between the sheriff’s office and the DA’s office or we can send it to the DA’s office and then they don’t send it to the defense. So then it comes up, well, we haven’t received all the discovery, and that can cause discovery violations in court, which eventually can lead to the case being dismissed because they’re not following the rules of evidence. Yeah. So that we’ve had a couple of cases with that issue where we I think I can think of two cases off the top of my head that horses had to be returned because there were three violations on the DA’s part. So the defense moved to dismiss the case and return the animals to probably a really bad situation. And we can’t do anything about that. Yeah. It’s just very frustrating.”*

[Investigator—BAP staff/agent, F, #9]

*“And that’s really frustrating for me, is that we file a lot of cases and those cases based on whatever it is, legislation, law, personal opinions, they end up going away.”*

[Law enforcement officer—sheriff, M, #19]

*“But I feel like there’s a lot of things that are happening in that mid-level of the DA’s office that these cases are just getting dropped. And they’re good, solid cases. It just seems like it’s the trend anymore is that these cases we’re getting are getting dropped or significantly plea bargain down.”*

[Law enforcement officer—sheriff, M, #19]

*“They [i.e., DAs] don’t look at it like everyone else does. It’s like, Oh, dammit, now I got to deal with this. I’m just going to get rid of it, dump it right away, or, you know, we’ll do the bare minimum. So that’s why I’m saying they need some education on it. And it needs to be more than] an hour training. Well, they need to have some extensive training. And we all do.”*

[Law enforcement officer, M, #11]

#### 3.1.3. Lack of Knowledge on How to Address Cases and Documentation Required

Law enforcement agents also commonly discussed a lack of knowledge and experience with animal cruelty and neglect, especially with regard to documentation (e.g., cost of care, what to put in warrants) and understanding what constitutes cruelty and neglect for different species.

*“And when we got involved in the case, there was some barriers with people not knowing how to do a large scale case and not having any idea what to look for, not having an idea of what to put in warrants to make the case move forward with the district attorney’s office.”*

[Investigator—BAP staff/agent, F, #1]

*“So there’s like, like me and my patrol sergeant, like we grew up ranch kids and, you know, so we know a lot about animals and what their condition should look like and so forth. Right. But the rest of my guys, like, they don’t they don’t have that knowledge. And you can do trainings and stuff like that. And we do those. But, you know, that really, really only goes so far. I mean, when you start talking about animal condition and neglect and stuff like that, like unless it’s something obvious, like no food, no water, no shelter, then they don’t know what they’re looking at.”*

[Law enforcement officer—sheriff, M, #15]

*“But we decided not to charge her because we had no expert to say what is a pig supposed to look like? And we don’t. Unless it’s a local farmer.”*

[Law enforcement officer/leadership, M/M, #21]

#### 3.1.4. Lack of Personnel to Conduct Investigations

Another common barrier discussed was having insufficient personnel to conduct thorough investigations on cases. While many law enforcement officers wanted to be able to address all animal cruelty/neglect cases, they discussed not having enough personnel to perform timely initial checks or follow-ups on calls, especially in rural areas.

*“[Law enforcement agency], they have three people on their staff right now. Three deputies. And […} they’re all on jury trial today, so they don’t have anybody on the street all week. So imagine what they’re going to be coming back to. They don’t have the time to deal with these cases.”*

[Investigator—BAP staff/agent, F, #1]

*“Our biggest barrier is just that we’re so busy, so sometimes follow ups, we struggle for a month and it could be a month or two weeks before we get there.”*

[Law enforcement officer, M, #12]

*“Every single year our biggest picture challenge right now is staffing. We’re eight people down and that makes such a huge impact on our ability to […] just to drive around […] investigate because, yeah, you know, we’ll get a call that’s 60 miles away. And we need both people that are on duty […] Because we don’t usually have an abundance of folks. So staffing right now. And I think that’s probably true of a lot of smaller sheriff’s offices throughout the state.”*

[Law enforcement officer/leadership, M/M, #22]

### 3.2. Additional Resource Needs for More Effectively Addressing Cases

The most commonly discussed additional resource needs to more effectively address cases included (1) resources for removing, transporting, housing, and caring for animals coming from cases; (2) more training for law enforcement and district attorneys on how to address cases; (3) access to expert animal investigators from BAP and CHS to serve as expert witnesses; and (4) access to social workers/mental health co-responders and other resources to address the underlying drivers of these cases.

Resources for removing, transporting, housing, and caring for animals coming from cases were the most commonly discussed need. Respondents discussed the need for access to trailers and other vehicles for transport, a centralized facility or certified rescues to take livestock to, and funds for care of animals.

*“Well, the transport and a place to transport them is always useful because we don’t have that.”*

[Law enforcement officer/leadership, M/M, #22]

*“Definitely the most helpful at the moment would be transportation and housing, especially for livestock.”*

[Law enforcement officer, M, #12]

Many respondents also discussed training needs for law enforcement and district attorneys on laws, procedures, and how to recognize animal cruelty/neglect for different species.

*“So the last time you guys came out, we had all every one of our deputies went through your guys’ training. And that helped tremendously. Since then, we have had a lot of turnover. And I don’t think any of our deputies that we still have on staff. Maybe there’s 3 or 4 that are still around that went through that. So I think we definitely need that training again. I think that’d be highly beneficial.”*

[Law enforcement officer, M, #13]

*“So I think that, you know, the more training, the more understanding law enforcement can [have] about animal neglect stuff, the better off those cases are going to get ran.”*

[Law enforcement officer—sheriff, M, #15]

Respondents also discussed the need for access to animal investigators who can serve as expert witnesses in the courtroom and help build cases.

*“I believe that the expert investigators is the most vital tool for us in our area. It’s just going to be so beneficial. And again, their testimony, if it goes to court, those are the things that are going to be the most beneficial for us, because if we testify on it, they’re going to ask us, what’s our training and experience? Well, that’s not going to be very much.”*

[Law enforcement officer—sheriff, M, #14]

*“I think an investigator that would come out and kind of go with us and get eyes on things because you guys see this way more often than we do. I think having someone that’s versed in training [who] specializes in that is huge whenever it comes to us.”*

[Law enforcement officer, M, #13]

Many law enforcement respondents recognized the role of mental health and other difficult life circumstances in driving animal cruelty/neglect, and the need for resources to address these challenges. Thus, respondents commonly discussed how access to a social worker or other mental health support for animal-related cases would be a beneficial resource.

*“So I would love to see [utilization of a social worker], you know, become a statewide movement so that all of the law enforcement agencies have somebody within their agency that can provide that level of support on a case when needed, de-escalation and whatnot.”*

[Investigator—BAP staff/agent, F, #4]

*“You know, animal abuse and animal mistreatment doesn’t happen in a vacuum. And very often these individuals are struggling with their own personal issues and […] they need support themselves. And so I think that’s a huge problem for a lot of cases. And also kind of ties into like maybe those cases aren’t maybe criminal enforcement is not the best option for those cases, but in order to access like really any of the services that are available for free, like through the state, they have to be charged and they have to enter the system that way, which is it creates a challenge and kind of a conflict of interest, you know, because, like, I don’t want to charge an 80 year old woman with dementia, with animal cruelty, like, I don’t want to do that, but I also don’t want her animals to like, be neglected.”*

[Investigator—BAP staff/agent, F, #7]

### 3.3. Emotional Toll

Numerous statements made by participants when sharing their challenges in handling animal abuse and neglect cases reflected significant emotional struggles including moral injury, helplessness, and frustration. For example, several agents voiced internal struggles when forced to return animals to dangerous situations due to legal or systemic constraints:

*“And those nine dogs were, by the condition, score of 1 or 2. And they had severe medical issues. We didn’t take anything that wasn’t, like, literally close to death. I mean, they were in bad, bad condition. And the judge ordered because he didn’t see anywhere in the statute that said he couldn’t. The dogs to go back during the court process. And so we had to return those dogs because of what the judge said.”*

[Investigator—BAP staff/agent, F, #1]

*“And we left. We left a large amount of animals on that property that were in bad condition because we were given parameters of what we could take… there was a lot of very upset individuals, including most of the staff at the sheriff’s office and all of our veterinarians and everybody involved in that case that we left animals there that were in really bad condition.”*

[Investigator—BAP staff/agent, F, #1]

Other agents talked about feelings of helplessness when working with vulnerable populations or those with mental health issues.

*“She had severe mental health problems… her fingernails were overgrown. Her toenails were overgrown. They were growing into her skin. She was covered in feces and urine… she desperately needed help… and when I left, I’m sure she still didn’t get any help.”*

[Investigator—BAP staff/agent, F, #9]

*“Unfortunately, you know, when somebody is struggling with severe mental health, it’s their kids or their animals or themselves and mostly suffer…we can go in, we can remove the animals… But that person is still struggling with mental health and that part is not getting met.”*

[Investigator—BAP staff/agent, F, #9]

*“He was very torn because, you know, just losing his wife, you know, those cats were their kids, their children. But, I mean, he didn’t have money to take care of them.”*

[Law enforcement officer, F/M, #10]

Many quotes reflected the frustration with trying to work within a problematic system and the personal toll and sacrifices made.

*“It’s undoubtedly the most stressful thing and traumatic thing that I’ve ever been to… I mean, you know, the human component is one thing, but for the most part, you’re talking to somebody if they’re still alive. But with that, I mean, it absolutely wrecked us.”*

[Law enforcement officer, M, #2]

*“I think they realize that they just aren’t doing anything about it. Yeah, it’s a failure. It’s an absolute failure of the system to address the issue.”*

[Law enforcement officer, M, #2]

*“He keeps dragging it out and dragging it out and dragging it out… He just doesn’t want to face the music. And he hasn’t had to, which is really I mean, it’s the animals that are suffering.”*

[Law enforcement officer/leadership, M/M, #22]

*“And I paid for the vet bill for myself, which was 3 or $400 out of my own pockets. But I’m an animal guy.”*

[Law enforcement officer, M, #13]

*“I took those dogs and I. I ended up bringing them to my own house and put them in the backyard until we could find something.”* ]

[Law enforcement officer/leadership, M/M, #21

This frustration included feelings of powerlessness, especially around repeat offenders:

*“…we can take this dog, we can take this horse, and then two weeks later, they get another one and they do the same thing. And we’re not really stopping the problem. We’re just removing the problem and hoping that they’ll learn and they they’re just not equipped to.”*

[Investigator—BAP staff/agent, F, #8]

*“It’s like a leper, people. They don’t all of a sudden start treating their animals. It just doesn’t happen. It’s just that they’re busy. They’re this. They’re that. They don’t have the money. They don’t… and the animal ends up suffering for it.”*

[Law enforcement officer/leadership, M/M, #22]

## 4. Discussion

Colorado has a comprehensive, state-run program to enforce the state’s cruelty laws around animal cruelty and neglect, including the Bureau of Animal Protection (BAP) program, yet our interviews with law enforcement and BAP agents identified several challenges including a lack of places to take removed animals, challenging relationships with district attorneys, a lack of adequate training, and a need for more expert animal investigators and social workers/mental health co-responders. Based on these results, we recommend the following courses of action, which could be implemented in Colorado or other states to address common challenges identified in this research.

### 4.1. Expand the Capacity for Housing Animals (Especially Livestock) Coming from Cases of Cruelty/Neglect

One of the biggest barriers facing law enforcement and the BAP is a lack of places to take animals coming from cruelty/neglect cases, especially livestock in need of critical veterinary care and/or quarantine. A state-run impound facility, or a group of state-certified facilities, could help address this problem by ensuring there is a safe place for animals to receive the care they need. Additionally, our findings suggest that law enforcement could benefit from grant funds to help purchase equipment for animal removal/transport and pay the costs of removal, temporary housing, and care of animals coming from cruelty/neglect cases.

### 4.2. Increase Access to Expert Investigators to Help Manage Criminal Cases, Serve as Expert Witnesses, and Help Remove Animals

Our findings suggest that law enforcement officers want to be able to address animal cases, but often lack the knowledge, personnel, and resources to do so. Our interviewees highlighted the importance of expert animal investigators to ensure cases are effectively addressed. Most law enforcement officers, even those with some animal training, are unable to adequately serve as expert witnesses for cruelty/neglect cases in court. Thus, if cases are to be successfully addressed throughout the state, it is critical that jurisdictions without an expert animal control officer have access to experienced non-profit and state BAP agents who can assist with all aspects of a case, including serving as an expert investigator/witness. Such expert investigators are often provided to law enforcement in other states via non-profit organizations; for example, in California, non-profit spcaLA Humane Officers may exercise the powers of a peace officer anywhere in the state while investigating animal cruelty [33], and in Oregon, Human Special Agents employed by the Humane Society are also sworn police officers with authority to investigate allegations of animal cruelty crimes [32].

### 4.3. Expand Training Opportunities for Law Enforcement, District Attorneys, and Judges on Animal Cruelty/Neglect Cases

While BAP and CHS provide basic training to law enforcement officers on how to address animal cruelty/neglect, our interviewees indicated a need for additional training for law enforcement/animal control officers as well as district attorneys and judges. Despite these educational efforts, many law enforcement agencies and agents do not feel that animal abuse is within their sphere of responsibility or feel they are low-priority calls [13]. This may be even more pronounced in communities with animal sheltering or control organizations. It has been suggested that this is similar to crimes against vulnerable persons, where family service-type organizations call the police, but are still expected to take a leading role [13]. Research suggests that law enforcement officers who are responsible for the enforcement of animal cruelty laws could benefit from having more knowledge of laws and more robust policies and procedures to follow [38]. This is a role that the BAP, non-profits employing BAP agents, and district attorney organizations can fill. Part of the equation for successful animal protection includes the involvement of prosecuting attorneys. To this end, the Association of Prosecuting Attorneys recognizes animal cruelty and fighting not only as precursor crimes to family and interpersonal violent crime, but also violent crime that should be effectively prosecuted [39]. It has also developed a statement of principles regarding the prosecution of animal cruelty crimes and continues to provide a national technical assistance network and produce a quarterly newsletter, the Lex Canis [39].

### 4.4. Create a State-Wide Position to Advise District Attorneys

Many law enforcement officers discussed challenges they face with working with district attorneys on animal cruelty/neglect cases. A state-wide animal cruelty special attorney position could address these challenges by serving in the role of mentoring, training, and assisting district and county attorneys with animal cruelty/neglect cases. For example, the state of Oregon allocates funds from the state budget to maintain the Animal Cruelty Deputy District Attorney (AC-DDA) role and make it a permanent program within the Department of Justice in Oregon [40]. The position was first created in 2013 when the Animal Legal Defense Fund provided funding that enabled Oregon to become the first U.S. state with a special prosecutor dedicated to pursuing animal-related cases.

### 4.5. Expand Opportunities for Forensic Training for Veterinarians

Our results suggest that having an expert veterinarian on cases is often critical for case success, and that the BAP veterinarian and veterinarians provided by CHS have been extremely helpful in assisting with cases where local veterinarians are unable to provide support. Interviewees voiced their belief that more cases could be successful if there were more veterinarians with veterinary forensics training to assist with cases. To address this need, we suggest that states partner with universities to develop coursework and/or a certification on veterinary forensics that would increase the number of veterinarians or even veterinary technicians able to serve as expert witnesses in court. In Oregon, the Oregon Humane Society addresses this need by providing the services of a forensic veterinarian and animal crime forensics center [32] dedicated to these types of cases.

### 4.6. Increase Mental Health/Social Work Support on Cases

Many law enforcement officers interviewed shared their belief that mental health challenges and difficult life circumstances are at the root of a significant number of animal cruelty/neglect cases. In fact, nearly every individual interviewed believed that more mental health resources would be beneficial. However, most officers reported having very little to no resources to address the mental health component of these cases. To ensure that the root causes of animal cruelty/neglect are being addressed, states could provide social workers who could accompany law enforcement agents on cases, perform case follow-up with animal owners, and ensure those in difficult life circumstances obtain the resources they need. There is a growing number of programs focused on training students in the field of veterinary social work [41], which could lead to more social workers available to assist with animal cruelty cases. The Oregon Humane Society, for example, hired a veterinary social worker, whose roles include connecting pet owners with social services including mental health counseling, access to pet food, health care, and other needs [42].

### 4.7. Amend State Statutes to Better Address Different Types of Cruelty/Neglect Situations Encountered

Many interviewees discussed the need to better address, define, and differentiate types of animal cruelty and neglect and ensure that charges match the type of cruelty/neglect occurring. For example, law enforcement officers mentioned how the statute could more directly address animal collecting, lack of timely veterinary care, animal fighting, and what differentiates standard livestock husbandry practices from cruelty. A stakeholder process with district attorneys, law enforcement, BAP, and others could be assembled to outline changes in the statute that would better identify, define, and differentiate the types of animal cruelty and neglect encountered throughout the state.

### 4.8. Increase Civil Capacity and/or Revise State Statutes to Make It Easier to Keep Animals Away from Perpetrators of Cruelty/Neglect

Numerous law enforcement officers discussed how they believe the ultimate goal of addressing animal cruelty/neglect cases should be to keep animals away from people who are perpetrators of cruelty/neglect, rather than jail time or fines. Yet the criminal system is not designed to achieve this goal. In Colorado, the Bureau of Animal Protection has the authority to use civil remedies such as administrative warrants and cease-and-desist orders and run civil cases to remove people’s animals if there is sufficient evidence of mistreatment without filing criminal charges. When the Bureau of Animal Protection has won such civil cases, the end result has been to prevent individuals from owning animals in the state. More resources aimed at these civil approaches and including such civil remedies in other state statutes could achieve this goal and help prevent people who have engaged in cruelty/neglect from owning animals.

These suggested changes are not only important for animal welfare, but also impact the welfare of those who care for these animals. The foundation of the One Welfare concept is a “collaborative approach for integrating animal welfare, human well-being and the environment” [43] (p. 12). Particularly relevant to the current study, the One Welfare framework acknowledges the link between animal and human abuse/neglect and that poor well-being in one domain contributes to poor well-being in the other. Seizures and surrenders, for example, are directly related to the poor well-being of the animal, but typically also include the poor well-being of the owner [44,45].

To address the needs of all involved, we suggest that in addition to the systemic changes suggested above, that organizations utilize a trauma-informed practice model. The use of trauma-informed practices benefit all those involved in animal welfare and protection, including professionals, animal owners, and the animals [20]. A trauma-informed model delivers services in a way that is appropriate and sensitive to the unique needs of each case [46]. At the foundation of a trauma-informed model is a basic understanding of the psychological and social impact that trauma can have on staff, persons accessing services, and others involved in the system, and responds by incorporating knowledge about trauma into their policies and procedures [46,47].

Law enforcement officers involved with animal abuse/neglect cases work with both animals and people who have experienced severe trauma [48], and as identified in our interviews, this work often results in feelings of helplessness, sadness, and frustration. The removal and surrender of animals are two examples of times when animal welfare workers often observe both animal and human suffering. These incidents take an emotional toll, often resulting in negative effects on workers’ well-being including exhaustion, depression, substance use, reduced well-being, sadness, feelings of guilt and anger, poor work satisfaction, and increased absenteeism [20,25,27]. Furthermore, compassion fatigue and burnout can negatively affect how animal welfare workers interact with people in the community, impacting workers’ ability to provide an emotionally safe environment, potentially leading to further trauma for the animal(s) and people involved [49].

There are many facets of animal welfare work that can contribute to burnout, including difficult working conditions, lack of training and support, poor management, lack of funding and local legislation, and public apathy [15,17,50]. The main symptoms of burnout are emotional and physical exhaustion, cynicism, a reduced sense of accomplishment, and feelings of inadequacy [51]. Burnout has been identified in numerous helping professions, including law enforcement [21,22,52,53]. One unique stressor for animal protection officers is the fact that many people underestimate and undervalue their work [15]. Other challenges include witnessing cases of extreme cruelty and personal threats [17]. These stressors, paired with workplace stressors such as large caseloads, emergency calls, aggressive animals, and challenging interactions with pet owners [15,17], place animal welfare workers at high risk for both compassion fatigue and burnout [15,17].

Trauma-informed models can help those involved in animal welfare build resilience and be better able to handle the challenges involved with regular exposure to trauma. To this end, trauma-informed approaches prioritize personnel’s emotional safety and incorporate policies that support comprehensive well-being through staff education, coaching/mentoring, and activities that support self-care [48,54]. There are six key components of trauma-informed models including safety; trustworthiness and transparency; peer support; collaboration and mutuality; empowerment, voice and choice; and cultural, historical, and gender issues [55].

Implementation of these key components includes the following:

- Ensuring physical and emotional safety;
- Creating a predictable work environment and clearly communicating boundaries;
- Building trust;
- Practicing transparency by being open and honest in communication, following through on commitments, and explaining the rationale behind decisions made;
- Peer support;
- Encouraging and creating opportunities for peer connections and mutual support;
- Collaboration and mutuality;
- Demonstrating the willingness to listen to all team members and respect their views and opinions;
- Empowerment, voice, and choice;
- Providing opportunities for all team members to make their own decisions when possible and demonstrate respect for their autonomy;
- Cultural, historical, and gender issues;
- Demonstrating the willingness to try and understand the unique experiences of all team members, including their diverse backgrounds, and consider cultural sensitivities when making decisions.

Benefits of a trauma-informed approach, including better case outcomes and positive impacts on staff, have been noted in numerous fields including police work, child welfare and protective services, and domestic violence services [19,46,50,54,56,57]. It is crucial to the success of a trauma-informed approach that the burden for mitigating burnout and compassion fatigue not be placed entirely on the worker [58]. The key to the successful implementation of a trauma-informed approach is substantial organizational change and support, including relevant policies and resources, and appropriate training and education [52,56,59]. A trauma-informed approach is a constantly evolving, multi-pronged, dynamic process. In order to create a more trauma-informed organization, multiple polices need to be implemented and employed in conjunction, including mental health supports, practices that recognize and mitigate secondary trauma, and positive leadership modeling [23,52,58,59,60]. For example, organizations could make social workers or mental health providers with specialized training in compassion fatigue and burnout, along with an understanding of animal welfare work, available to employees.

This study has several limitations that should be noted. The interviews were all conducted with law enforcement and BAP agents within the state of Colorado, so caution should be taken when generalizing to other agencies and organizations outside of Colorado. Responding honestly involved participants trusting that their identity would remain confidential, and some may have been reluctant to share all of their concerns from a fear of breached confidentiality. While a prior working relationship with one of the authors (BN) and a BAP agent assisting with this study was useful in obtaining interviews, it may have inadvertently impacted responses. In addition, it is possible that the individuals who chose to be interviewed were not representative of all law enforcement officers and agents, even within the state of Colorado. Future quantitative research could examine the quantity of animal cruelty cases and the types of cases law enforcement officers and agents manage, and how barriers to addressing cases may vary based on these factors.

## 5. Conclusions

In conclusion, the results of this study highlight areas for potential change in addressing animal cruelty and neglect cases, offering insights into possible best practices. Suggested changes include expansion of temporary housing options, additional training, expert investigators and social workers/mental health professionals, and possible amendments to state statutes. In addition, the use of a trauma-informed model is recommended to best meet the psychological needs of all those involved in animal welfare work. Together, these changes can help organizations successfully address animal abuse and neglect cases, for the betterment of law enforcement officers, pet owners, the public, and arguably most important—abused and neglected animals.

## Figures and Tables

**Table 1:** Participant role and gender.

Role	Gender	Participant Number
Investigator—BAP staff/agent	F	1
Law enforcement officer	M	2
Law enforcement officer/leadership	M	3
Investigator—BAP staff/agent	F	4
Law enforcement officer—sheriff	M	5
Law enforcement officer	F	6
Investigator—BAP staff/agent	F	7
Investigator—BAP staff/agent	F	8
Investigator—BAP staff/agent	F	9
Law enforcement officer	F/M	10
Law enforcement officer	M	11
Law enforcement officer	M	12
Law enforcement officer	M	13
Law enforcement officer—sheriff	M	14
Law enforcement officer—sheriff	M	15
Law enforcement officer—sheriff	M	16
Law enforcement officer—sheriff	M	17
Law enforcement officer—sheriff	M	18
Law enforcement officer—sheriff	M	19
Law enforcement officer—sheriff	M	20
Law enforcement officer/leadership	M	21
Law enforcement officer/leadership	M	22
Law enforcement officer—sheriff	M	23
Law enforcement officer—undersheriff	M	24

## Data Availability

Data available upon request.

## Supplementary Materials

The following supporting information can be downloaded at: https://www.mdpi.com/article/10.3390/ani14243602/s1, S1. Brief description of Colorado’s civil and criminal laws to address animal cruelty and neglect; S2. Interview Questions.
