# Supplementary material for: Animal Abuse Investigations: Challenges and Recommendations to Improve Animal and Human Welfare
Source: Animals (Basel). 2024 Dec 13;14(24):3602. doi: 10.3390/ani14243602 (PMC11672660; doi:10.3390/ani14243602)
Supplement: Supplementary file 1 [file animals-14-03602-s001.zip › animals-3344268-supplementary.pdf]

Supplemental materials

S1. Brief description of Colorado's civil and criminal laws to address animal cruelty and neglect

Colorado has both civil and criminal laws to address animal cruelty and neglect. On the civil side, Article 42, Title 35 of the Colorado Revised Statutes states that: "No animal shall be mistreated or neglected to such degree or abandoned in any circumstance so that the animal's life or health is endangered." This act also gives Bureau of Animal Protection (BAP) agents and the Colorado Department of Agriculture authority to enforce Colorado's animal protection statute by conducting investigations related to animal mistreatment and issuing summons and complaints, typically in partnership with local law enforcement. This act also provides the Colorado Department of Agriculture Commissioner (and thus the Bureau of Animal Protection staff) with the authority to issue a cease-and-desist order, seek temporary or permanent injunctive relief to enforce compliance with the Act, and take charge of, provide for, or remove any companion animal, as well as any livestock animal pursuant to court order, found to be mistreated so that the animal's life or health is endangered.

Colorado's criminal code, codified in title 18 of the Colorado Revised Statutes, provides that a person commits cruelty to animals if he or she "knowingly, recklessly or with criminal negligence overdrives, overloads, overworks, torments, deprives of necessary sustenance, unnecessarily or cruelly beats, allows to be housed in a manner that results in chronic or repeated serious physical harm, carries or confines in or upon any vehicles in a cruel or reckless manner, engages in a sexual act with an animal, or otherwise mistreats or neglects any animal or causes or procures it to be done, or having the charge or custody of any animal, fails to provide with proper food, drink, or protection from the weather consistent with the species, breed, and type of animal involved, or abandons an animal." Section 18-9-202(1)(a), C.R.S. states that "a person commits cruelty to animals if he or she recklessly or with criminal negligence tortures, needlessly mutilates, or needlessly kills an animal" (section 18-9-202(1.5)(a), C.R.S.) or "intentionally abandons a dog or cat." Section 18-2-202(b), C.R.S.

While the above laws apply to both companion and livestock animals, Colorado's laws also clarify that "acceptable husbandry practices" for livestock are exempt from animal cruelty laws (C.R.S. § 18-9-201.5.). Furthermore, while all BAP agents, including non-profit agents such as those employed by the Colorado Humane Society, can investigate complaints related to companion animals, only local law enforcement and BAP agents employed by the Colorado Department of Agriculture can investigate complaints related to livestock.

S2

Interview Questions

- Tell me about an animal cruelty/neglect case you were working on that you think was effectively addressed. What made that possible?
- Tell me about an animal cruelty/neglect case you were working on that you think was not effectively addressed. What happened, and what was the key barrier?
- Tell me about a case that was called into you that you decided to not pursue. Why did you decide not to pursue it?
- Have you had any challenges working with district attorneys on cruelty/neglect cases? If so, what challenges? What about your experience with judges in your district?
- What collaborations or resources have been really helpful to you in addressing cruelty cases in your jurisdiction?
- When you do an investigation of alleged cruelty/neglect and you don't find sufficient evidence of cruelty/neglect yet but you do have some concerns, how often do you issue warnings, provide education to an owner, and/or go back out for follow up visits to make sure treatment for an animal improves? What are some of the barriers to this kind of follow up?
- Have you ever had to return animals to an owner in a case where you believe you gathered sufficient evidence to demonstrate animal cruelty/neglect was occurring? If so, what happened?
- What generally happens to companion animals when there is a cruelty investigation? When are they removed, and to where? What about horses or livestock animals?
- Have you ever left animals on the property in a case where you believe you gathered sufficient evidence to demonstrate animal cruelty/neglect was occurring? If so, why?
- How often do animal-related cases involve mental health challenges or other difficult life circumstances? Do you have resources available, such as social workers or mental health co-responders, who can go out on these cases with you?
- There are a variety of types of resources or support that are or could be potentially available to help you in addressing animal cruelty/neglect cases. For example, trainings for law enforcement/DAs, expert investigators or veterinarians provided by non-profits or the state, assistance with animal removal, facilities to take animals, social workers, etc). Which resources do you already use and find helpful? What resources would be most helpful for you?
- How familiar are you and your officers with the elements of the state animal cruelty statute? If you could make one change to the current statute to be able to more effectively address animal cruelty/neglect in your jurisdiction, what would it be?
